# Carvacrol-Based Formulations Modulate Sirtuins and Exert Cytotoxic and Antimicrobial Effects in Lung Cancer Models

**DOI:** 10.3390/antiox15060719

**Published:** 2026-06-05

**Authors:** Selin Aktar Kiremitci, Ayşe Simay Metin, İmren Hasoğlu, Mert Geçim, Didem Demir, Emine Hande Karagedik, Pınar Yurdakul Mesutoğlu, Öykü Gönül Geyik

**Affiliations:** 1 Pharmaceutical Botany Department, Faculty of Pharmacy, Istinye University, 34408 Istanbul, Türkiye; selin.aktar@istinye.edu.tr; 2 Medical Biology and Genetics Doctoral Program, Institute of Graduate Education, Istinye University, 34408 Istanbul, Türkiye; 3 Department of Biochemistry, Faculty of Pharmacy, Istinye University, 34408 Istanbul, Türkiye; 4 Department of Biochemistry, Institute of Health Sciences, Marmara University, 34865 Istanbul, Türkiye; 5 Department of Medical Microbiology, Faculty of Medicine, Istinye University, 34408 Istanbul, Türkiye; 6 Department of Medical Laboratory Techniques, Vocational School, Işık University, 34980 Istanbul, Türkiye; 7 Medical Biology Department, Faculty of Medicine, Istinye University, 34408 Istanbul, Türkiye

**Keywords:** essential oil, NSCLC, A549, SIRT1, chorioallantoic membrane assay, antimicrobial susceptibility testing

## Abstract

Lung cancer remains a leading cause of cancer-related mortality and is frequently complicated by respiratory infections, supporting interest in agents with both antitumoral and antimicrobial potential. This study evaluated two standardized thyme-derived, carvacrol-based formulations, Vacrol and S-Mix, in lung cancer-associated experimental models. A549 lung adenocarcinoma and BEAS-2B bronchial epithelial cells were treated with the formulations, and cell viability, clonogenic capacity, SIRT1–SIRT7 protein expression, in ovo tumor growth, histopathological changes, and antimicrobial activity against pneumonia-associated reference strains were assessed. S-Mix showed stronger short-term cytotoxicity in A549 cells, reaching an IC_50_ of 1 mM after 72 h, whereas Vacrol produced more pronounced modulation of selected sirtuin proteins, particularly SIRT1, SIRT4, and SIRT5. Both formulations suppressed colony formation under prolonged exposure. In the CAM model, Vacrol was associated with greater macroscopic suppression of tumor growth and vascularization, while S-Mix produced more prominent histopathological cellular injury. Vacrol also showed antimicrobial activity against tested respiratory pathogens, with MIC values ranging from 0.5 to 4 mg/mL, MBC values ranging from 1 to 4 mg/mL and volatile-phase activity against *Streptococcus pneumoniae* as well as *Klebsiella pneumoniae*. These findings suggest that carvacrol-based formulations exert distinct cytotoxic, sirtuin-modulatory, antitumoral, and antimicrobial effects, warranting further mechanistic and translational validation.

## 1. Introduction

Lung cancer is the leading cause of cancer incidence and cancer-related death worldwide, with approximately 2.48 million new cases and over 1.8 million deaths recorded in 2022 [1,2]. Molecular diagnostics, targeted therapies, and immunotherapy have improved outcomes in some patient subgroups, but survival in advanced-stage disease remains poor [3]. This has motivated a search for complementary strategies that address tumor cell survival alongside the metabolic adaptations and clinical complications, particularly infection and chronic inflammation, that accompany lung malignancy [3,4].

Pulmonary infections, particularly pneumonia, are a specific threat in lung cancer patients. Tissue disruption compromised mucosal barriers, immune dysregulation, and the immunosuppressive effects of anticancer treatments all raise susceptibility to pneumonia [4,5]. Recurrent or unresolved pulmonary infections may, in turn, sustain chronic inflammation, tissue injury, and microenvironmental alterations that are increasingly recognized as contributors to lung carcinogenesis and poorer clinical outcomes [4,6,7]. The bacteria most isolated from lung cancer patients with pneumonia are Gram-negative species, especially *Klebsiella pneumoniae* and *Pseudomonas aeruginosa*, *though Staphylococcus* spp., *Streptococcus* spp., and *Haemophilus influenzae*, and *Enterobacter* spp. also recur frequently [8]. Among these, *K. pneumoniae* and *P. aeruginosa* infections have been linked through specific molecular pathways to lung cancer progression [8].

Of particular concern, these pathogens are frequently multidrug-resistant, which further complicates clinical management. Given the increasing burden of antimicrobial resistance, the limited effectiveness of conventional therapies against pneumonia-associated pathogens in lung cancer patients has driven increasing interest in alternative antimicrobial strategies. This overlap between malignancy and infection has prompted interest in agents that combine antitumoral and antimicrobial activity, especially against pathogens commonly found in lung cultures of cancer patients [4,5].

One of these agents is carvacrol, a phenolic monoterpene found in the essential oils of *Thymus* and *Origanum* species, which interferes with mitochondrial homeostasis and has shown inhibitory effects on tumor cell proliferation and pro-apoptotic activity in A549 lung adenocarcinoma cells [9,10]. Carvacrol and carvacrol-rich preparations also inhibit clinically relevant bacterial pathogens, making them potentially useful where malignancy and infection overlap [10,11,12]. The biological potency of carvacrol-based formulations may vary depending on the accompanying volatile constituents and phytochemical profile, which together can affect membrane permeability, redox balance, and cellular stress responses [9,13].

These cellular responses are closely linked to sirtuins, a family of NAD^+^-dependent deacylases that regulate energy metabolism, oxidative stress responses, mitochondrial integrity, genomic stability, and cell survival. In non-small cell lung cancer, individual sirtuins can function as either tumor promoters or tumor suppressors depending on the cellular context [14,15]. The mitochondrial members SIRT3, SIRT4, and SIRT5 are directly involved in metabolic reprogramming, redox regulation, and mitochondrial adaptation, processes that support cancer cell resilience [14,16]. Dysregulation of sirtuin signaling in lung cancer has been linked to unchecked proliferation, resistance to apoptosis, and altered metabolic fitness, and selective modulation of these proteins could represent a viable therapeutic approach [15,16].

In this context, carvacrol-based formulations represent plausible candidates for further investigation. Among these formulations, Vacrol and S-Mix come forward as standardized thyme-derived volatile formulations with different phytochemical profiles [17]. Vacrol is enriched in carvacrol, while S-Mix has a broader terpene composition that includes α-pinene, limonene, and 1,8-cineole alongside carvacrol [17]. A carvacrol-dominant formulation may favor more direct cytotoxic and mitochondria-associated effects, whereas a compositionally diverse mixture may engage broader pathways, though these distinctions need experimental confirmation.

To investigate these formulation-dependent effects, we evaluated the cytotoxic, clonogenic, sirtuin-modulatory, in ovo antitumoral, and antimicrobial activities of Vacrol and S-Mix in lung cancer-associated experimental models. Cytotoxic and antiproliferative effects were assessed in A549 lung adenocarcinoma cells and BEAS-2B non-tumorigenic bronchial epithelial cells using the SRB viability assay and colony formation assay. Changes in SIRT1–SIRT7 protein expression were measured by enzyme-linked immunosorbent assay (ELISA), antitumoral activity was assessed using the chorioallantoic membrane (CAM) assay, and antimicrobial activity was evaluated against pneumonia-associated bacterial pathogens using antimicrobial susceptibility testing. Notably, the combination of antitumoral and antimicrobial activities within a single plant-derived formulation is of particular clinical relevance in lung cancer patients, who face elevated susceptibility to pulmonary infections and multidrug-resistant pathogens.

We hypothesized that the distinct phytochemical profiles of Vacrol and S-Mix would result in differential biological responses, with the carvacrol-enriched formulation showing more pronounced sirtuin-modulatory and in ovo antitumoral effects.

## 2. Materials and Methods

### 2.1. Cell Culture

The A549 (CRM-CCL-185) and BEAS-2B (CRL-3588) (ATCC, Manassas, VA, USA) cell lines were used as experimental models. A549 is a human epithelial lung carcinoma cell line, and BEAS-2B is a non-tumorigenic human bronchial epithelial cell line. Both cell lines were cultured in RPMI-1640 supplemented with 10% fetal bovine serum (FBS), 1% L-glutamine, and 1% penicillin/streptomycin (Gibco, Thermo Fisher Scientific, Waltham, MA, USA), and maintained in a humidified 5% CO_2_ incubator at 37 °C as described previously [18].

### 2.2. Treatments

Vacrol and S-Mix are standardized thyme-derived volatile formulations previously characterized by GC-MS [17]. According to the reported compositional analysis, Vacrol contains a higher proportion of carvacrol, whereas S-Mix exhibits a broader terpene profile. In addition to carvacrol, the major constituents identified in these formulations included α-pinene, 1,8-cineole, limonene, linalool, eugenol, and cinnamaldehyde, with S-Mix being relatively enriched in α-pinene, limonene, and 1,8-cineole [17]. These compositional differences were considered when interpreting the biological responses observed following treatment.

### 2.3. Cell Viability Assay

The effect of Vacrol and S-Mix on cell viability was determined using the sulforhodamine B (SRB) assay. A549 cells were seeded into 96-well plates in 100 µL of medium at densities of 5 × 10^3^, 3 × 10^3^, and 2 × 10^3^ cells/well for 24, 48, and 72 h timepoints, respectively. BEAS-2B cells were seeded at 2 × 10^3^ cells/well for the 72 h timepoint only.

After 24 h of incubation, A549 cells were treated with Vacrol and S-Mix at concentrations of 1 nM, 10 nM, 100 nM, 1 µM, 10 µM, 100 µM, 1 mM, 2 mM, 2.5 mM, 5 mM, and 10 mM. Carvacrol at 100 µM was applied as a positive control. Stock concentrations were prepared based on Trolox equivalents (Vacrol: 10.848 mM; S-Mix: 4.236 mM). BEAS-2B cells were treated at the IC_50_ concentration determined for each formulation. Cells were incubated for 24, 48, or 72 h, then fixed with 50% (*w*/*v*) trichloroacetic acid (TCA). Following fixation, TCA was removed with distilled water, SRB solution was added, and plates were incubated in the dark. Excess SRB was removed with 1% acetic acid, and plates were air-dried. Bound dye was solubilized with 150 µL/well of 10 mM Tris base (pH 10.0). Absorbance was measured at 564 nm using a microplate reader (BMG Labtech, LUMIStar Omega, Ortenberg, Germany). Each condition was performed with 6 technical replicates across 3 independent replicates (n = 3).

### 2.4. Colony Formation Assay

A549 and BEAS-2B cells were seeded in 6-well plates at densities of 500 and 750 cells per well, respectively, and allowed to adhere overnight. Cells were then treated with 1 mM Vacrol or 1 mM S-Mix for 72 h. Following treatment, the medium was removed and cells were washed twice with 1× PBS. Cells were fixed in ice-cold absolute methanol for 15 min and subsequently stained with 0.2% (*w*/*v*) crystal violet for 30 min. After removing excess dye with distilled water, plates were allowed to dry at room temperature. The experiment was continued for an average of 10–14 days and terminated when colonies of at least 50 cells were observed in control groups. Clusters of 50 or more cells were scored as colonies.

### 2.5. Protein Extraction

A549 and BEAS-2B cells were seeded into 6-well culture plates at a density of 2 × 10^5^ cells per well and incubated for 24 h. Subsequently, cells were treated with 1 mM Vacrol or 1 mM S-Mix for 72 h. After incubation, cells were detached by trypsinization and washed twice with ice-cold PBS. Protein extraction was carried out using RIPA buffer (EcoTech Biotechnology, Erzurum, Türkiye) according to the manufacturer’s instructions. Cell lysates were centrifuged at 13,000 rpm for 10 min at 4 °C, and supernatants were collected for further analysis.

### 2.6. Quantification of Sirtuin Protein Expression by ELISA

The expression levels of sirtuin family members (SIRT1–SIRT7) were quantitatively determined using commercial sandwich ELISA kits (BT Lab, Shanghai, China; Cat. Nos: #E2557Hu, #E2558Hu, #E2559Hu, #E2560Hu, #E6506Hu, #E2562Hu, #E2233Hu) according to the manufacturer’s protocols. Briefly, standards and samples were added to microplates pre-coated with specific capture antibodies. Following the addition of biotinylated detection antibodies and streptavidin-HRP conjugate, plates were incubated for 1 h at 37 °C. After five consecutive washes, substrate solution was added and plates were incubated in the dark for 10 min at 37 °C. The reaction was terminated with stop solution, and optical density (OD) was measured at 450 nm using a microplate reader (LUMIStar Omega, BMG Labtech, Ortenberg, Germany). All procedures were performed across three independent biological replicates (n = 3). Protein concentrations were calculated from standard curves and normalized to the non-treated control group, expressed as percentage of control.

### 2.7. Chorioallantoic Membrane (CAM) Assay

The antitumoral effects of Vacrol and S-Mix were evaluated using the chorioallantoic membrane (CAM) assay [19]. Fertilized Ross 308 chicken eggs were incubated at 37 °C with 60% relative humidity. On embryonic development day (EDD) 1, small holes were drilled into the lateral and lower (air sac-containing) parts of the shell to allow air entry and membrane detachment, and the eggs were placed in the incubator with the pointed end facing upward. On EDD4, a 1–2 cm circular window was opened on the upper surface of the eggshell, sealed with adhesive tape, and the eggs were returned to the incubator. Embryo viability was monitored daily throughout the experiment. On EDD7, A549 cells (1 × 10^6^) were mixed with Matrigel (Corning, Corning, NY, USA) and applied onto the chorioallantoic membrane. On EDD10, 50 µL of Vacrol or S-Mix, calculated based on Trolox values and diluted accordingly in Hanks’ Balanced Salt Solution (HBSS), was applied directly onto the tumors, which were photographed. On EDD13, tumors were re-photographed, excised from the CAM, and fixed in 10% buffered formalin. Fixed samples were processed in a semi-open carousel-type tissue processor. Tissues were dehydrated in graded alcohols (50%, 70%, 80%, 96%, and 100%), cleared with xylene, and embedded in paraffin at 58–60 °C. Paraffin blocks were sectioned at 3–5 µm using a rotary microtome (CUT 5062, Slee, Nieder-Olm, Germany). Sections were stained with hematoxylin and eosin (H&E) for histopathological evaluation. For each experimental condition, approximately 10 fertilized eggs were initially grafted with tumor cells. Only viable embryos at EDD10 were included in subsequent analyses. At least two tumor-bearing embryos per group were analyzed histologically and macroscopically. Tumor size was quantitatively evaluated from macroscopic images using ImageJ software (version 1.54, NIH, Bethesda, MD, USA). To reduce variability related to image magnification and zoom differences, measurements were normalized based on the diameter of a prominent adjacent blood vessel visible in each image. Representative images presented in the figures were selected for visualization purposes only.

### 2.8. Antimicrobial Susceptibility Testing

*Methicillin Resistant Staphylococcus aureus* (MRSA) ATCC 43300, *Streptococcus pyogenes* ATCC 12344 *(S. pyogenes)*, *Streptococcus pneumoniae* ATCC 49619 (*S. pneumoniae*), and *Klebsiella pneumoniae* ATCC BAA-1144 (*K. pneumoniae*) were used as ATCC reference strains. The minimum inhibitory concentration (MIC) as well as the minimum bactericidal concentration (MBC) values were used to evaluate the antibacterial efficacy, and MIC/MBC values were determined using the broth microdilution method according to EUCAST guidelines [20]. Vacrol was used as the CF and tested alone for antimicrobial susceptibility testing, as its carvacrol content was higher than that of S-Mix.

Effect of two-fold serial dilutions (9–1.125 mg/mL) of CF against the pathogenic strains were initially screened, followed by lower concentrations from 2 to 0.0039 mg/mL. Volatile-phase antimicrobial activity of CF against *S. pneumoniae* and *K. pneumoniae* was tested with disc volatilization assay, using 0.040 mg/mL CF embedded discs placed on the lid of the Petri dishes.

### 2.9. Statistical Analysis

All statistical analyses were performed using GraphPad Prism v.9 software (GraphPad Software, San Diego, CA, USA). For cell viability data, statistical differences between groups were assessed using the Kruskal–Wallis test followed by Dunn’s multiple comparisons test. Tumor volume data obtained from the CAM assay were also analyzed using the Kruskal–Wallis test followed by Dunn’s multiple comparisons test. For sirtuin protein expression data, a two-way analysis of variance (ANOVA) was performed to evaluate the main effects of cell line and treatment, as well as their interaction, followed by Tukey’s multiple comparisons test. Data are presented as mean ± SEM for ELISA and cell viability experiments, and as mean ± SD for CAM assay experiments. A *p*-value of <0.05 was considered statistically significant.

## 3. Results

### 3.1. S-Mix Is More Cytotoxic than Vacrol in A549 Cells

**Figure 1:**
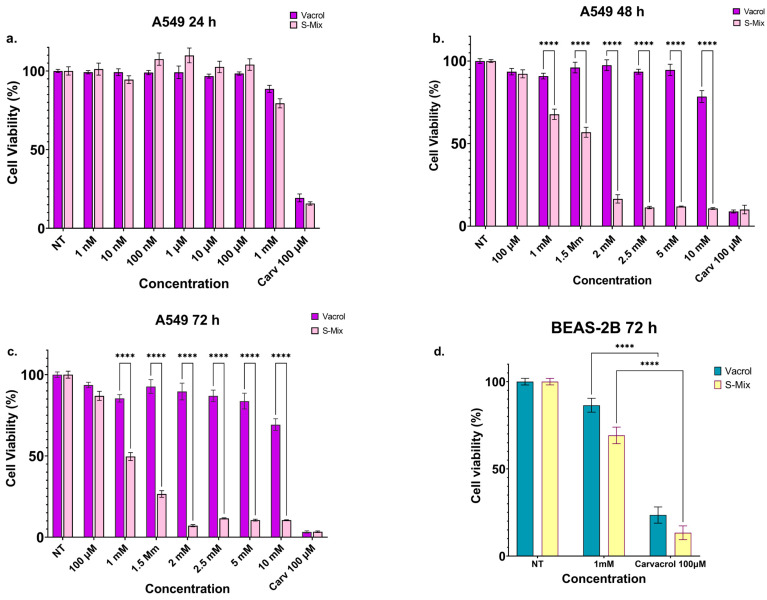
Effects of S-Mix and Vacrol on the viability of A549 lung cancer cells after (**a**) 24 h, (**b**) 48 h, and (**c**) 72 h of treatment. (**d**) Effects of S-Mix and Vacrol on the viability of BEAS-2B non-tumorigenic lung cells after 72 h of treatment at 1 mM (IC_50_) concentration. Data are presented as mean ± SEM of 3 biological and 6 technical replicates (n = 3). Significant differences are indicated by asterisks (**** *p* < 0.0001; Kruskal–Wallis followed by Dunn’s multiple comparisons test).

The effects of Vacrol and S-Mix formulations on the cell viability of the A549 lung cancer cell line were evaluated using the SRB assay, with carvacrol serving as the positive control (Figure 1a–c). In preliminary screenings, carvacrol reduced cell viability by only 3% at 100 µM; therefore, higher concentrations of carvacrol were not included in the study. This decision was based on the well-established solubility limitations of carvacrol in aqueous culture media at higher concentrations, and on the study objective of comparing the two standardized formulations rather than characterizing free carvacrol as a primary test agent. Carvacrol at 100 µM served as a reference control, consistent with previously published data in A549 cells [21]. S-Mix suppressed cell viability in a concentration- and time-dependent manner significantly more effectively than Vacrol. Following 72 h of incubation, the IC_50_ of S-Mix was determined to be 1 mM, whereas cell viability in Vacrol-treated cells at the same concentration remained as high as 85.4%. Even at the highest tested concentration of 10 mM, Vacrol reduced cell viability by only 69.2%.

To assess selectivity, BEAS-2B non-tumorigenic bronchial epithelial cells were treated with the IC_50_ concentrations of each formulation for 72 h. Cell viability was 86.4% in the Vacrol group and 69.1% in the S-Mix group. In the carvacrol positive control group, viability was 23.5% for Vacrol and 13.5% for S-Mix (Figure 1d). These results indicate that both formulations exert selective cytotoxic effects on cancer cells relative to non-tumorigenic cells, with S-Mix demonstrating significantly greater biological activity than either free carvacrol or Vacrol.

### 3.2. Vacrol and S-Mix Abolish Colony Formation in Both Cell Lines

**Figure 2:**
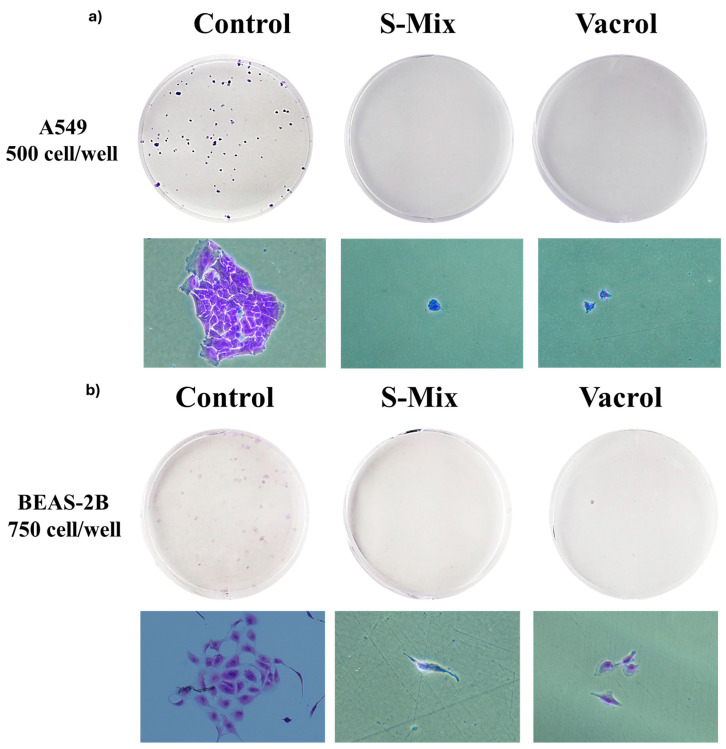
Colony formation images of A549 and BEAS-2B cells. Microscope images of (**a**) A549 lung cancer cells and (**b**) BEAS-2B non-tumorigenic bronchial epithelial cells following treatment with IC_50_ concentrations of Vacrol and S-Mix (Magnification 10×).

A549 cells were seeded at 500 cells per well and BEAS-2B cells at 750 cells per well. Vacrol and S-Mix were applied at IC_50_ concentrations for an average of 10–14 days. The experiment was terminated when colonies of at least 50 cells were observed in control groups. As shown in Figure 2, colony formation was observed exclusively in control groups for both cell lines. No colony formation was detected in cells treated with either S-Mix or Vacrol, indicating that both formulations completely abolish the clonogenic potential of A549 and BEAS-2B cells at IC_50_ concentrations.

### 3.3. Vacrol Targets Mitochondrial Sirtuins SIRT1, SIRT4, and SIRT5

**Figure 3:**
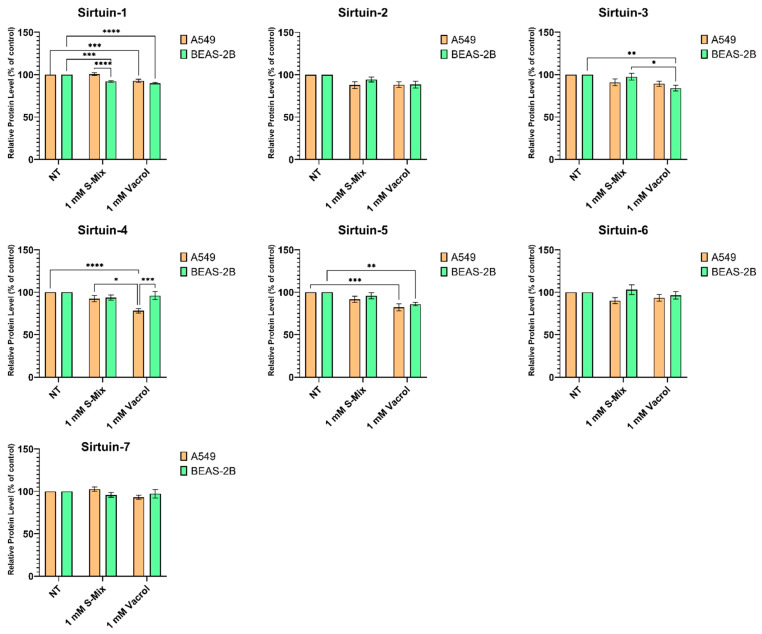
Effects of S-Mix and Vacrol treatment on sirtuin protein expression in A549 and BEAS-2B cell lines. Cells were treated with 1 mM S-Mix or 1 mM Vacrol for 72 h. Protein levels of SIRT1–SIRT7 were determined by sandwich ELISA and normalized to the non-treated control group (% of control). Data are presented as mean ± SEM of three independent experiments (n = 3); two-way ANOVA with Tukey’s post hoc test. SIRT1: A549 NT vs. Vacrol (*** *p* = 0.0009); BEAS-2B NT vs. S-Mix (*** *p* = 0.0003), NT vs. Vacrol (**** *p* < 0.0001). SIRT3: BEAS-2B S-Mix vs. Vacrol (* *p* = 0.0282), NT vs. Vacrol (** *p* = 0.0049). SIRT4: A549 S-Mix vs. Vacrol (* *p* = 0.0133); A549 Vacrol vs. BEAS-2B Vacrol (*** *p* = 0.0010); NT vs. Vacrol (**** *p* < 0.0001). SIRT5: A549 NT vs. Vacrol (*** *p* = 0.0005); BEAS-2B NT vs. Vacrol (** *p* = 0.0099).

To evaluate the effects of Vacrol and S-Mix on the cellular sirtuin profile, A549 and BEAS-2B cells were treated with 1 mM of each formulation for 72 h, and SIRT1–SIRT7 protein levels were determined by sandwich ELISA (Figure 3). Overall, Vacrol exerted a pronounced suppressive effect on specific sirtuin isotypes.

Analysis of SIRT1 expression revealed that Vacrol treatment significantly reduced protein levels in both A549 (*p* = 0.0009) and BEAS-2B (*p* < 0.0001) cells compared to the non-treated (NT) control group. S-Mix treatment also correlates with a significant decrease in SIRT1 levels, but exclusively in BEAS-2B cells (*p* = 0.0003).

SIRT3 and SIRT4 protein levels exhibited cell line- and agent-specific responses. In BEAS-2B cells, Vacrol significantly decreased SIRT3 levels compared to both the NT control (*p* = 0.0049) and the S-Mix-treated group (*p* = 0.0282). Regarding SIRT4, Vacrol demonstrated a strong inhibitory effect in A549 cells, which was statistically significant compared to both the NT control (*p* < 0.0001) and S-Mix groups (*p* = 0.0133). Importantly, the suppressive effect of Vacrol on SIRT4 was significantly more pronounced in A549 cells than in BEAS-2B cells (*p* = 0.0010).

Vacrol treatment significantly downregulated SIRT5 in both cell lines, reducing levels compared to the NT group in A549 (*p* = 0.0005) and BEAS-2B (*p* = 0.0099) cells. In contrast, no statistically significant differences were observed in SIRT2, SIRT6, or SIRT7 protein expression between groups or cell lines following treatment with either agent. Taken together, these findings demonstrate that Vacrol possesses potent and selective downregulatory activity, particularly targeting the protein expression levels of SIRT1, SIRT4, and SIRT5.

### 3.4. Vacrol Reduces Tumor Growth and Vascularization in the CAM Model

The in ovo antitumoral effects of Vacrol and S-Mix were assessed using the chorioallantoic membrane (CAM) assay. A549 cells were implanted onto the CAM of fertilized chicken eggs, and tumor development was monitored following treatment with Vacrol (1 mM) or S-Mix (1 mM).

**Figure 4:**
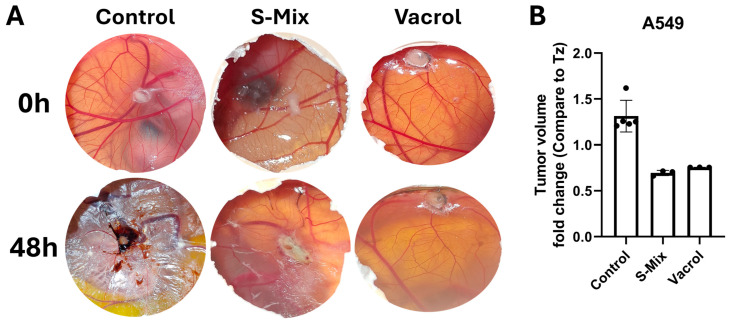
(**A**) Macroscopic images of CAM assay specimens. A549 lung cancer cells were implanted onto the chorioallantoic membrane and treated with Vacrol (1 mM) or S-Mix (1 mM). Upper panel shows representative images from individual eggs within each group; lower panel shows additional replicates. Non-treated control eggs display prominent tumor masses with extensive neovascularization. (**B**) S-Mix-treated eggs show reduced tumor size and decreased vascular density. Vacrol-treated eggs exhibit the most pronounced suppression of both tumor growth and tumor-associated angiogenesis.

As shown in Figure 4, macroscopic examination of CAM specimens revealed differences in tumor morphology and vascularization among the experimental groups. In the non-treated control group, tumor masses with neovascularization and branching blood vessel networks were observed surrounding the implantation site. In eggs treated with S-Mix, tumor size appeared smaller and the density of newly formed blood vessels converging toward the tumor was visibly reduced compared to the control. In Vacrol-treated eggs, tumor formation appeared smaller and fewer blood vessels were observed converging toward the implantation site compared to the other groups. Both formulations reduced tumor growth in the CAM model, with a greater macroscopic reduction in the Vacrol group.

**Figure 5:**
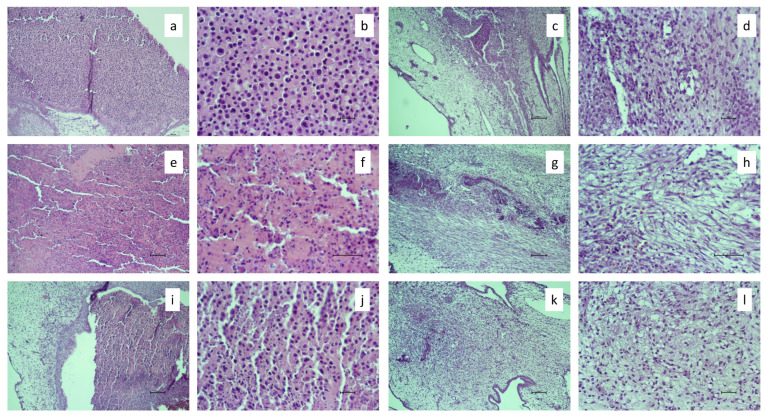
Histopathological examination images of in ovo CAM tissue following Vacrol and S-Mix administration. (**a**,**b**) A549-Control at 10× and 40× magnification showing tumor cells within the Matrigel matrix. (**c**,**d**) Adjacent CAM tissue of control group at 10× and 40×, showing no apparent histopathological changes. (**e**,**f**) A549-S-Mix at 10× and 40×, demonstrating disrupted cell morphology and cellular disorganization suggestive of cell injury and apoptosis. (**g**,**h**) Adjacent CAM tissue of S-Mix group at 10× and 40×, showing swollen nucleated erythrocytes with stomatocyte-like morphology. (**i**,**j**) A549-Vacrol at 10× and 40×, showing increased cellular injury compared to control but relatively less pronounced than S-Mix. (**k**,**l**) Adjacent CAM tissue of Vacrol group at 10× and 40×, with no apparent histopathological alterations. Sections were stained with hematoxylin and eosin (H&E).

Histopathological evaluation of CAM tissues (Figure 5) showed a different pattern. In the control group (a, b), A549 cells seeded within the Matrigel matrix were clearly identifiable, and the adjacent CAM tissues (c, d) showed no apparent histopathological alterations. In the S-Mix-treated group (e, f), disruption of cell morphology and cellular disorganization were observed compared to the control, which may be indicative of cell injury, cell death, or apoptotic changes. In the CAM tissues adjacent to S-Mix-treated Matrigel (g, h), no overt histopathological changes were noted; however, nucleated erythrocytes appeared swollen with a stomatocyte-like morphology, possibly indicating cellular damage. In the Vacrol-treated group (i, j), increased cellular injury was observed compared to the control, although the degree of morphological disruption appeared less pronounced than that seen in the S-Mix group (e, f). Adjacent CAM tissues in the Vacrol group (k, l) showed no apparent histopathological alterations. Histopathological findings ranked the degree of cellular injury as S-Mix > Vacrol > Control.

### 3.5. Vacrol Shows Antimicrobial Activity Against Pneumonia-Associated Pathogens

**Figure 6:**
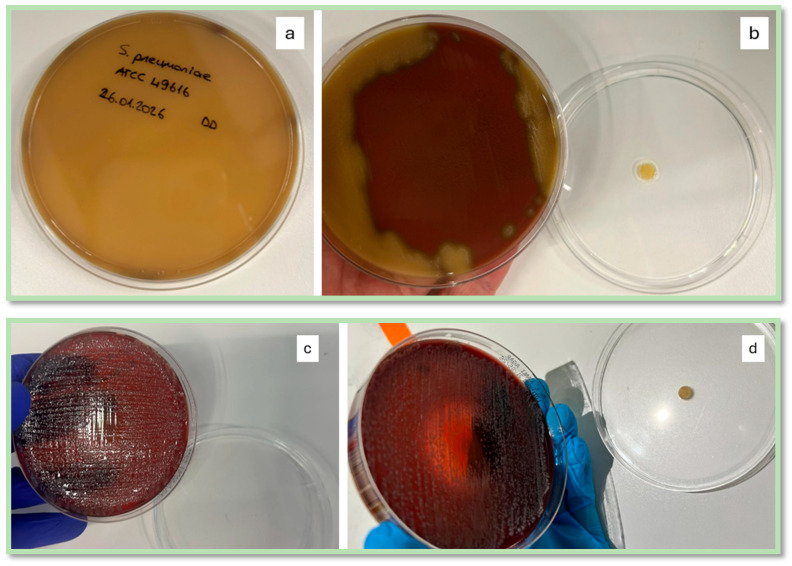
(**a**) *Streptococcus pneumoniae* ATCC 49619 growth control plate, (**b**) volatile-phase antimicrobial activity of the carvacrol formulation (CF) against *Streptococcus pneumoniae* ATCC 49619, (**c**) *Klebsiella pneumoniae* ATCC BAA-1144 growth control plate, and (**d**) volatile-phase antimicrobial activity of CF against *Klebsiella pneumoniae* ATCC BAA-1144.

Selective antimicrobial/bactericidal activity of Vacrol was investigated against all the pathogens tested. MIC values were found to be 0.5 mg/mL for *S. pneumoniae*, 1 mg/mL for MRSA and *S. pyogenes*, and 2 mg/mL for *K. pneumoniae*. MBC values were found to be 2 mg/mL for *K. pneumoniae*, 1 mg/mL for *S. pyogenes*, 4 mg/mL for MRSA, and 2 mg/mL for *S. pneumoniae* as depicted in Table 1. Notably, volatile-phase exposure of CF completely suppressed the visible growth of *S. pneumoniae*, whereas only a limited inhibitory effect was observed against *K. pneumoniae.* (Figure 6b and Figure 6d, respectively). Control plates demonstrated marked growth (Figure 6a and Figure 6c, respectively).

## 4. Discussion

We investigated the effects of S-Mix and Vacrol on sirtuin family proteins (SIRT1–7) in A549 lung adenocarcinoma and BEAS-2B normal bronchial epithelial cells and tested the antimicrobial activity of Vacrol against pneumonia-associated pathogens. Vacrol showed a strong inhibitory effect on specific sirtuin isotypes, namely SIRT1, SIRT4, and SIRT5, while S-Mix had a more limited impact.

Sirtuins regulate cellular metabolism, stress response, and tumorigenesis, and their dysregulation is frequently implicated in cancer progression [22,23]. The targeted downregulation of these proteins by Vacrol suggests a previously undescribed mechanism underlying its biological activity.

Carvacrol, the primary active compound in our formulation, is a potent monoterpene previously shown to induce cell cycle arrest, elevate reactive oxygen species (ROS) production, and trigger mitochondrial dysfunction-mediated apoptosis in A549 cells [23,24]. While the intrinsic anticancer properties of isolated carvacrol are well-documented, our data indicate that the Vacrol formulation exhibits enhanced therapeutic efficacy. According to our compositional analysis, Vacrol is a complex plant-derived essential oil formulation containing 50.1% carvacrol, complemented by other bioactive phytochemicals such as 1,8-cineole, linalool, eugenol, and cinnamaldehyde [17]. The superior bioactivity of Vacrol compared to free carvacrol can be attributed to synergistic interactions among these components, which collectively facilitate cellular membrane penetration and act on multiple molecular targets simultaneously.

An important observation is the difference in potency between the two formulations across different assay types. In the SRB viability assay, S-Mix was substantially more cytotoxic than Vacrol (S-Mix achieved an IC_50_ of 1mM, whereas Vacrol did not reach an IC_50_ within the tested concentration range, maintaining viability above 50% even at 10 mM). However, Vacrol was the stronger sirtuin modulator and showed greater macroscopic antitumoral activity in the CAM model. This apparent paradox likely reflect different mechanisms of action: the broader terpene profile of S-Mix (enriched in α-pinene, limonene, and 1,8-cineole) appears to produce a more general cytotoxic effect through multiple cellular targets, whereas the carvacrol-enriched composition of Vacrol may act more selectively on mitochondrial pathways, including sirtuin regulation. The distinction between short-term cytotoxicity and mechanism-specific activity is relevant for therapeutic development, as target-selective agents may offer better safety profiles than broadly cytotoxic compounds.

Specifically, the profound suppression of SIRT1 by Vacrol in both A549 and BEAS-2B cells is of particular importance. SIRT1 is largely characterized as a tumor promoter in NSCLC, promoting cell survival, proliferation, and drug resistance [25,26]. The inhibition of SIRT1 allows pro-apoptotic targets such as p53 and FOXO to remain in an acetylated (active) state, thereby driving cancer cells toward apoptosis [27]. S-Mix also reduced SIRT1 levels, but only in BEAS-2B cells, a cell-type-specific difference that needs further investigation.

The differential regulation of SIRT4 between A549 and BEAS-2B cells following Vacrol treatment is a notable finding of this study. Vacrol suppressed SIRT4 expression in A549 cells to a significantly greater degree than in BEAS-2B cells. SIRT4 is a mitochondrial sirtuin that has been characterized as a tumor suppressor in several cancer types, repressing glutamine metabolism and maintaining genomic stability [27,28]. Its downregulation by Vacrol may therefore seem counterintuitive. However, the role of sirtuins in cancer is context-dependent, and the same sirtuin can act as either a tumor promoter or a tumor suppressor depending on the cancer type, cellular context, and microenvironment [14,21]. In A549 cells, the simultaneous suppression of SIRT1 (a tumor promoter) and SIRT4 alongside SIRT5 may create a combined metabolic stress that overwhelms the cancer cell’s adaptive capacity, even if SIRT4 loss alone would be expected to favor tumor growth. While this net biological outcome of multi-sirtuin expression modulation remains a hypothesis-generating interpretation that warrants detailed functional validation (such as metabolic flux or genetic knockdown/overexpression studies), it provides a plausible conceptual framework consistent with the observed antitumoral activity of Vacrol in the CAM model and is supported by the concept that disruption of mitochondrial sirtuin networks as a whole can shift the balance toward cell death rather than adaptation.

Vacrol also significantly downregulated SIRT5 in both cell lines and SIRT3 in BEAS-2B cells. Since SIRT3, SIRT4, and SIRT5 are primarily mitochondrial, their collective suppression by Vacrol suggests a potential link to altered mitochondrial dynamics, although further functional assays are required to confirm this. The lack of statistically significant changes in the expression of SIRT2, SIRT6, and SIRT7 suggests a degree of target specificity, while the downregulation of mitochondrial sirtuin expression levels points toward a potential involvement of cellular energy dynamics and oxidative stress management [29,30,31]. However, it is important to note that the current study evaluated sirtuin alterations solely at the protein expression level via ELISA. Consequently, these findings reflect changes in protein abundance rather than direct changes in sirtuin enzymatic activity or definitive functional disruption of downstream mitochondrial pathways.

In the colony formation assay, neither Vacrol nor S-Mix allowed colony formation at IC_50_ concentrations in either A549 or BEAS-2B cells. While the complete suppression of clonogenic capacity in A549 cancer cells is a desirable outcome, the absence of colony formation in BEAS-2B cells raises a question about selectivity. However, this finding should be interpreted in the context of assay duration and the fact that BEAS-2B cell line is a non-tumorigenic human bronchial epithelial model [32,33]. The SRB assay measures short-term viability over 72 h, where BEAS-2B cells maintained 86.4% (Vacrol) and 69.1% (S-Mix) viability. The colony formation assay, by contrast, evaluates long-term proliferative capacity over 10–14 days of continuous exposure. The difference between preserved short-term viability and abolished long-term clonogenic potential suggests that these formulations impair the proliferative machinery of normal cells upon prolonged exposure. As acknowledged, this long-term antiproliferative effect on non-tumorigenic cells poses a potential toxicity concern, and the translational implications of these findings must be approached with caution. Whether this in vitro effect translates to clinical toxicity in vivo—where cells are subject to different pharmacokinetic clearance rather than continuous, concentration-dependent exposure—requires strict evaluation in future models.

The CAM assay results reinforced the in vitro findings. Both Vacrol and S-Mix reduced tumor growth, but Vacrol produced a greater macroscopic reduction in tumor size and vascularization. Histopathological evaluation, however, ranked the degree of cellular injury as S-Mix > Vacrol > Control. This discordance between macroscopic and histopathological assessments is not unexpected in the CAM model, where macroscopic tumor size can be influenced by hematoma formation, stromal cell ingrowth from the CAM into the tumor mass, and fluid accumulation, factors that do not necessarily correlate with the degree of cellular damage observed histologically [34,35]. The two formulations therefore appear to act through partially different mechanisms: Vacrol may primarily suppress tumor vascularization and gross tumor expansion, while S-Mix appears to cause greater direct cellular injury at the microscopic level. These observations should be interpreted with caution, as histopathological findings in the CAM model can vary between biological replicates, and cells incubated in ovo may undergo physical changes during the experimental period. Beyond its antitumoral effects, Vacrol also demonstrated activity against pneumonia-associated bacterial pathogens, an important consideration given the high susceptibility of lung cancer patients to respiratory infections.

Pneumonia in lung cancer patients is frequently associated with bacterial and fungal pathogens, contributing to increased morbidity and mortality, particularly in the setting of multidrug-resistant (MDR) infections. While carvacrol-rich essential oils have demonstrated antimicrobial activity, systematic evaluations of standardized formulations remain limited [11,12,36]. We show here that Vacrol has antimicrobial activity against all isolates tested with MIC values ranging from 0.5 mg/mL (*S. pneumoniae*) to 2 mg/mL (*K. pneumoniae*) and MBC values ranging from 1 mg/mL to 4 mg/mL (Table 1). The volatile-phase activity against *S. pneumoniae* and *K. pneumoniae* is of particular interest because it raises the possibility of inhalation-based delivery, which could achieve local antimicrobial concentrations in the respiratory tract while minimizing systemic exposure. This restricted inhibitory effect suggest that higher concentrations may be required to achieve a more pronounced antibacterial effect against *K. pneumoniae*. Still, this remains speculative and would require pharmacokinetic and tolerability studies before any clinical consideration.

This study has several limitations. First, only one lung cancer cell line (A549) was used; validation in additional non-small cell lung cancer lines with different molecular backgrounds (e.g., H460, H1299) would strengthen the generalizability of the findings. Second, sirtuin expression was measured only at the protein level by ELISA; complementary analysis at the mRNA level (RT-qPCR) or measurement of enzymatic activity would provide a more complete picture of sirtuin modulation. Furthermore, because this study did not include functional mitochondrial assays, ROS measurements, apoptosis analyses, or genetic modulation (knockdown/overexpression) studies, the precise causal relationship between sirtuin downregulation and Vacrol-induced cytotoxicity remains to be fully elucidated. Third, the CAM model, while useful for initial screening, does not fully replicate the complexity of mammalian tumor biology; confirmation in murine xenograft models is necessary before drawing conclusions about in vivo relevance. Fourth, a limitation of the current study is that the antimicrobial evaluation was conducted using reference strains, which may restrict the immediate translational applicability of the findings to complex clinical settings. To address this, a dedicated research approach focused on testing multidrug-resistant clinical respiratory isolates has already been initiated, and these forthcoming clinical data will be presented in a subsequent study. Finally, the pharmacokinetic and toxicological profiles of Vacrol and S-Mix remain unknown, and these must be characterized before any translational consideration.

## 5. Conclusions

In this study, Vacrol selectively downregulated SIRT1, SIRT4, and SIRT5 in A549 lung adenocarcinoma cells while no statistically significant differences were observed in the expression of SIRT2, SIRT6, and SIRT7, suggesting a potential target specificity toward mitochondrial and nuclear sirtuin members. The differential expression profile of SIRT4 in A549 cells compared to BEAS-2B cells suggests a degree of selectivity that warrants further exploration. S-Mix, despite being more cytotoxic in the short-term viability assay, produced weaker sirtuin modulation, pointing to a mechanistic distinction between the two formulations. In the CAM model, Vacrol showed greater macroscopic tumor suppression, while S-Mix caused more histopathological cellular injury, reinforcing the idea that these formulations act through different pathways. Vacrol also exhibited broth-phase and volatile-phase antimicrobial activity against pneumonia-associated pathogens, with MIC values of 0.5–2 mg/mL and MBC values of 1 mg/mL to 4 mg/mL. Future studies should focus on clarifying the molecular mechanisms by which Vacrol regulates sirtuin expression, validating these findings in additional lung cancer cell lines and murine xenograft models, and evaluating potential synergistic interactions with conventional anticancer and antimicrobial agents.

## Figures and Tables

**Table 1:** Minimum Inhibitory Concentration (MIC) and Minimum Bactericidal Concentration (MBC) values of the carvacrol formulation (CF) against pneumonia-associated ATCC strains. MIC assays were performed by two-fold serial dilutions (2–0.0039 mg/mL). CF was dissolved in DMSO (final DMSO < 1%).

Test Condition	*K. pneumoniae*		*S. pyogenes*		MRSA		*S. pneumoniae*	
	MIC	MBC	MIC	MBC	MIC	MBC	MIC	MBC
CF	2 mg/mL	2 mg/mL	1 mg/mL	1 mg/mL	1 mg/mL	4 mg/mL	0.5 mg/mL	2 mg/mL
Vehicle control (DMSO < 1%)	Growth	Growth	Growth	Growth	Growth	Growth	Growth	Growth
Growth control	Growth	Growth	Growth	Growth	Growth	Growth	Growth	Growth
Medium control	No growth	No growth	No growth	No growth	No growth	No growth	No growth	No growth

MRSA, Methicillin-Resistant *Staphylococcus aureus*; CF, Carvacrol Formulation; MIC, Minimum Inhibitory Concentration; MBC, Minimum Bactericidal Concentration.

## Data Availability

The data supporting the findings of this study are available from the corresponding author upon request.

## Abbreviations

The following abbreviations are used in this manuscript:

A549	Human lung adenocarcinoma cell line
ANOVA	Analysis of variance
ATCC	American Type Culture Collection
BEAS-2B	Human bronchial epithelial cell line
CAM	Chorioallantoic membrane
DMSO	Dimethyl sulfoxide
EDD	Embryonic development day
ELISA	Enzyme-linked immunosorbent assay
EUCAST	European Committee on Antimicrobial Susceptibility Testing
FBS	Fetal bovine serum
GC-MS	Gas chromatography–mass spectrometry
HBSS	Hanks’ Balanced Salt Solution
H&E	Hematoxylin and eosin
IC_50_	Half-maximal inhibitory concentration
MBC	Minimum bactericidal concentration
MDR	Multidrug-resistant
MIC	Minimum inhibitory concentration
MRSA	Methicillin-resistant Staphylococcus aureus
NAD^+^	Nicotinamide adenine dinucleotide
NSCLC	Non-small cell lung cancer
OD	Optical density
PBS	Phosphate-buffered saline
RIPA	Radioimmunoprecipitation assay buffer
RPMI	Roswell Park Memorial Institute medium
SEM	Standard error of the mean
SD	Standard deviation
SIRT	Sirtuin
SRB	Sulforhodamine B
TCA	Trichloroacetic acid
